# Effects of Different 16-Week Exercise Interventions on Bone Mineral Density of Sedentary Older Women

**DOI:** 10.1093/geroni/igab046.3251

**Published:** 2021-12-17

**Authors:** Amy Ellis, Kristi Crowe-White, Gary Hunter

**Affiliations:** 1 University of Alabama, Tuscaloosa, Alabama, United States; 2 The University of Alabama, Tuscaloosa, Alabama, United States; 3 University of Alabama at Birmingham, Birmingham, Alabama, United States

## Abstract

Multicomponent exercise that includes both resistance and aerobic training is recommended to prevent loss of bone mineral density (BMD) in postmenopausal women. However, optimal training frequency has not been determined. Sixty-three non-osteoporotic sedentary women ages 60y and older were randomized to one of three exercise groups for sixteen weeks: 1) one resistance and one aerobic session per week, 2) two resistance and two aerobic sessions per week, or 3) three resistance and three aerobic sessions per week. Resistance exercise included supervised sessions on weight machines, and aerobic exercise was treadmill walking. BMD of the hip and lumbar spine (L1-L4) was assessed by dual energy X-ray absorptiometry (Prodigy, GE Medical Systems Lunar, Madison, WI, software version 6.10.029), and z scores were calculated from a reference population adjusted for age and sex. Among the total cohort with BMD measurements at week 16 (n=58; 83% white), z scores improved for the trochanter, Ward’s triangle, total hip, L1 and L4. Within-group comparisons showed improvement at the trochanter, total hip, and L1 for group 2 only, while only group 1 demonstrated an increase at L4 (p<0.05 for all). However, no time-by-group interactions were observed. Sixteen weeks of combined resistance and aerobic training is effective for improving BMD of older adult women. Results suggest training frequency of two sessions per week may be optimal. Postmenopausal women should be encouraged to do aerobic exercise such as walking plus resistance training at least once weekly to prevent osteoporosis.

